# Cannabinoid and cannabinoid related receptors in fibroblasts, inflammatory and endothelial cells of the equine hoof with and without laminitis: novel pharmacological target

**DOI:** 10.3389/fvets.2025.1723160

**Published:** 2025-11-28

**Authors:** Rodrigo Zamith Cunha, Francesca Gobbo, Maria Morini, Giulia Salamanca, Augusta Zannoni, Chiara Bernardini, Alessandro Gramenzi, Roberto Chiocchetti

**Affiliations:** 1 Department of Veterinary Medical Sciences, University of Bologna, Bologna, Italy; 2 Department of Veterinary Medicine, University of Teramo, Teramo, Italy; 3 Department of Translational Medicine and for Romagna, University of Ferrara, Ferrara, Italy

**Keywords:** endocannabinoid system, immune cells, macrophages, neutrophils, T-cells

## Abstract

**Background:**

Evidence suggests that the endocannabinoid system (ECS) is crucial for regulating inflammation, cell proliferation and pain. The ECS is composed of cannabinoid receptors such as type 1 (CBR1), type 2 (CBR2) and GPR55, endocannabinoids and enzymes. Proteins of ECS have previously been localized in the epidermal cells of the horse hooves. Given the physio-pathological role and cellular distribution of the ECS across species, the authors hypothesized that cannabinoid receptors are expressed within the inflammatory cells, fibroblasts and endothelial cells of the equine hoof laminae, going beyond the epidermal cells.

**Objectives:**

To preliminary analyze the gene expression of Cn1r, Cn2r and GPR55 in the hoof laminae and test the specificity of the antibody against GPR55. To characterize the distribution and expression of CBRs in the inflammatory cells and fibroblasts of the laminar junction of equine healthy hooves and with laminitis.

**Animals:**

Animals were divided into 3 groups: healthy, acute laminitis and chronic laminitis. A total of 18 samples were collected and processed from the front limb of animals slaughtered for consumption or euthanized (6 control animals, 4 acute laminitis, 8 chronic laminitis).

**Methods:**

Analysis of CBR1, CBR2 and GPR55 protein expression was made by fluorescence microscopy with co-localization with antibodies against the macrophages marker IBA1, the T cell marker CD3, the neutrophils marker calprotectin (MAC387), the fibroblasts marker vimentin (Clove V9) and the nerve fibers marker Substance P. Preliminary analysis was performed to evaluate gene expression (*Cnr1*, *Cnr2*, and *Gpr55*) using real-time PCR and to verify the specificity of the primary antibody (Gpr55) with Western Blotting (WB).

**Results:**

The resident pool of inflammatory cells in the normal laminae and the inflammatory infiltrate cells of the affected equine laminae showed protein expression of CB2R and GPR55; no CB1R staining was seen at the inflammatory cells. Equine dermal fibroblast and endothelial cells exhibited protein expressions of CB1R, CBR2 and GPR55. Substance P positive nerve fibers were positive for CB1R.

**Conclusions and clinical importance:**

Cannabinoid receptors are expressed in different immune cell types of the hoof laminae, pointing to the role of the ECS in modulating inflammatory outburst, tissue degeneration and pain. Our results serve as a foundation for the development of new veterinary pharmacotherapies that target the ECS during laminitis.

## 1 Introduction

Laminitis is a complex and devastating condition affecting the hooves of horses; it is characterized by inflammation and failure of the laminae, namely the structures which secure the distal phalanx to the hoof wall (1). The pathogenesis of the disease involves multiple factors, including metabolic and endocrine disorders, gastrointestinal disturbances, and systemic inflammatory responses (2, 3). Horses affected with Pars Pituitary Intermedia Disfunction (PPID) or Insulin Dysregulation (ID) commonly present with lamellar damage (2, 4).

Inflammation is a central feature of laminitis, involving various cellular and molecular pathways. Upon the onset of laminitis, an inflammatory cascade is triggered, leading to the activation and recruitment of several inflammatory cell types to the laminae (5). Macrophages and dendritic cells (DCs) are pivotal in orchestrating the inflammatory response (6). Neutrophils are among the first responders to the site of inflammation; are essential for pathogen clearance but can also cause significant collateral damage to the laminar tissue (7). A significant increase in the number of neutrophils and T-cells is noted in horses with induced acute laminitis which points to the role of this cell type in the onset of the disease (7, 8).

In addition to the fluctuation of inflammatory cell populations during the disease process, recent studies have shown that the recruitment of inflammatory cells and cytokine production are also influenced by environmental factors (9). Neutrophil and lymphocyte concentrations in peripheral blood display opposing patterns during daylight hours, whereas during the night both cell populations tend to converge. Interleukin-6 (IL-6) levels peak around 8 a.m., while at 8 p.m. they reach their lowest concentrations (9). This rhythmic fluctuation over a 24-h period is known as a circadian cycle. Similarly, endocannabinoid molecules also exhibit circadian oscillations, being largely responsible for regulating stress responses, sleep, and neuronal oxidative balance (10–12). It is therefore plausible to hypothesize that the connection between the immune system and environmental cues is mediated, at least in part, by the expression of endocannabinoid system proteins within inflammatory cells.

Recent studies have identified cannabinoid receptors in equine tissue, suggesting a role for the endocannabinoid system (ECS) in mediating responses to pain and inflammation (13–16). The ECS proteins have being identified in the epidermal laminae of horses with and without laminitis (17), placing in evidence the fluctuation of gene expression and protein during this disease. The ECS is an intricate signaling network within the body, acting as a critical regulator of the various physiological processes, including pain, inflammatory response and endocrine pathways, responsible for maintaining homeostasis (18). This system includes endocannabinoids, receptors, and enzymes, all working in concert to modulate metabolic functions, neurotransmission and immune responses (19).

While the ECS is a conserved system across species, there are notable variations which reflect the unique physiological needs of different organisms. Understanding these variations is crucial for tailoring therapeutic interventions. In equine medicine, the ECS has garnered particular attention due to its potential implications in managing pain and stress (20–22). Studies have recently shown the therapeutic benefits of using cannabinoids as the drug of choice in treating mechanical allodynia and behavior disorders in horses (23, 24), and in reducing inflammatory response (22, 25). Given the physio-pathological role and cellular distribution of the ECS across species, the authors hypothesized that cannabinoid receptors are also expressed within the inflammatory cells, fibroblasts and endothelial cells of the equine hoof laminae, going beyond the epidermal cells. Thus, the aim of this study is to verify the protein expression of cannabinoid receptors at the inflammatory cells present at the hoof laminae during the course of laminitis.

## 2 Materials and methods

### 2.1 Type of study

Observational, non-blinded *post-mortem* analytical, cross-sectional study with a comparative design.

### 2.2 Animals

Laminar samples were collected *post-mortem*. Tissue collection occurred strictly from animals either euthanized for humane reasons (hospital patients – private owned) or slaughtered for human consumption (slaughterhouse). A total of 18 samples were collected from different animals. The characteristics of the horses are presented in Table 1.

**Table 1:** Summary of the animals which samples were taken.

Groups	Age (Years)	Origin	BREED	SEX	Limb
1CTRL	1	SlaugtherHouse	French Trotter	F	LF
2CTRL	3	SlaugtherHouse	Trotter	M	LF
3CTRL	5	SlaugtherHouse	Mix	F	RF
4CTRL	3	SlaugtherHouse	Mix Trotter	M	LF
5CTRL	5	SlaugtherHouse	Mix	M	RF
6CTRL	7	SlaugtherHouse	Trotter	M	RF
1ALL	3	Private Owner	Throurobread	F	RF
2ALL	5	SlaugtherHouse	French Trotter	F	RF
3ALL	25	Private Owner	Warmblood	F	LF
4ALL	4	Private Owner	French Trotter	M	LF
1CLL	3	SlaugtherHouse	Trotter	M	RF
2CLL	8	SlaugtherHouse	Trotter	M	RF
3CLL	10	SlaugtherHouse	Mix	F	LF
4CLL	12	SlaugtherHouse	Haflinger	M	RF
5CLL	11	SlaugtherHouse	Trotter	F	RF
6CLL	9	Private Owner	Mix	F	LF
7CLL	17	SlaugtherHouse	Haflinger	M	RF
8CLL	5	Private Owner	Mix	F	LF

CTRL, Control Group; ALL, Acute Laminitic Laminae; CLL, Chronic Laminitic Laminae; LF, Left Front; RF, Right Front.

Healthy laminae group (control) - The hooves were collected from the thoracic limbs of 6 healthy horses slaughtered for consumption, with ages ranging from 1 to 7 years. All the hooves collected were macroscopically intact.

Laminitic laminae group - The hooves were collected from the thoracic limbs of 12 laminitic horses, with ages ranging from 3 to 25 years. The horses were subdivided in two subgroups: the acute laminitic (ALL) horses (4/12), and the chronic laminitic (CLL) horses (8/12).

### 2.3 Ethical statement

According to Directive 2010/63/EU of the European Parliament and of the Council of 22 September 2010 regarding the protection of animals used for scientific purposes, Italian legislation (D. Lgs. n. 26/2014) does not require any approval by competent authorities or ethics committees since this study did not influence any therapeutic decisions or induced any clinical outcomes. All samples were taken on demand after death. No disease was induced.

### 2.4 Group division

The samples were divided into three groups: Healthy Laminae (HL), Acute Laminitis (ALL), and Chronic Laminitis (CLL). Group division was based on a combination of clinical history, anamnesis, physical examination, and evidence of anatomical failure of the hoof (e.g., sinking of the third phalanx, rotation with severe angle, or excessive heel growth), as described (17).

Acute laminitis was defined as a clinical presentation occurring within a maximum of 72 h from the onset of clinical signs, with no prior history of laminitis and no evidence of structural failure (1, 26). Chronic laminitis was defined as a condition persisting for more than 72 h after the onset of primary clinical signs, with structural failure and/or a history of recurrent laminitic episodes (1, 27).

All cases were confirmed by *post-mortem* histopathological analysis carried out by a specialized pathologist, which included findings such as basement membrane separation, marked irregular hyperplasia of epidermal lamellae, distorted secondary lamellae, and inflammatory cell infiltration, as previously described (28). Detailed inclusion and exclusion criteria for each group are presented in Table 2.

**Table 2:** Summary of the inclusion and exclusion criteria of the sample’s group division.

Group	Inclusion criteria	Exlcusion criteria
CNTRL	Age between 1 and 10 years; Body weight ranging from 250 to 600 kg; Both sexes (male and female) and any breed; Clinically healthy at the time of selection;	Any evidence of lameness or orthopedic disorders; Presence of systemic comorbidities with clinical impact; History of laminitis or visible signs of hoof pathology; Pregnancy
ALL	Age between 1 and 25 years; Body weight ranging from 250 to 600 kg; Both sexes, any breed; Diagnosed with acute laminitis, defined as: Onset of clinical signs within 72 h; No prior known history of laminitis; Absence of structural failure (radiographically or macroscopically).	Previous know episodes of laminitis; Pre-existing anatomical hoof defects.
CLL	Age between 1 and 25 years; Body weight between 250 and 600 kg; Both sexes, any breed; Diagnosed with chronic laminitis, defined as: clinical condition persisting for more than 72 h after onset of initial signs; Associated with structural failure of the hoof and/or recurrent episodes of laminitis.	Not applicable beyond those outlined for acute cases, as all cases included demonstrated structural damage.

CNTRL, Control Group; ALL, Acute Laminitic Laminae; CLL, Chronic Laminitic Laminae.

### 2.5 Sample collection and tissue processing

The digits were disarticulated at the metacarpophalangeal joint. For each subject one limb was collected and the same limb was used for the experiments. Lamellar tissue, including epidermal and dermal lamellae was obtained by sectioning the hooves with a band saw according to the stablished protocol (Supplementary Figure 1) (17, 29, 30). All the tissues were collected a maximum of 4 h after death. For microscopic evaluation the tissue was then fixed and processed to obtain cryosection as described (17). The lamellar tissues were fixed for 48 h at 4 °C in 4% paraformaldehyde in phosphate buffer (0.1 M, pH 7.2). Tissues were subsequently rinsed overnight in phosphate-buffered saline (PBS; 0.15 M NaCl in 0.01 M sodium phosphate buffer, pH 7.2) and stored at 4 °C in PBS containing 30% sucrose and sodium azide (0.1%). The following day, the tissues were transferred to a mixture of PBS − 30% sucrose–azide and Optimal Cutting Temperature (OCT) compound (Sakura Finetek Europe, Alphen aan den Rijn, The Netherlands) at a ratio of 1:1 for an additional 24 h before being embedded in 100% OCT in Cryomold® (Sakura Finetek Europe). The sections were prepared by freezing the tissues in isopentane cooled in liquid nitrogen. Cryosections (14 μm thick) of lamellar tissues were cut on a cryostat and mounted on polilysinated slides, for each horse 5 non-consecutive slides (*n* = 5) were made for each co-localisation. Cryosections were oriented transverse to the major axis of the horse finger and included the primary (PEL) and secondary epidermal laminae (SEL), a portion of the innermost layer of the hoof capsule, and a large portion of the sub-lamellar dermal tissue.

In addition to the sampling for microscopic evaluation, aliquots of fixed and non-fixed tissue sample (frozen with liquid nitrogen) were harvested for gene expression (performed by RT-PCR) and western blotting data, respectively.

### 2.6 Analysis of gene expression by real-time polymerase chain reaction

**Figure 1:**
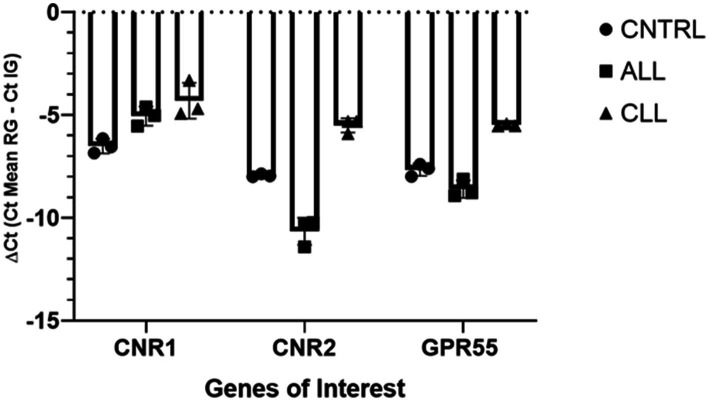
Gene expression of *Cnr1*, *Cnr2*, *GPR55*, in equine laminitic laminae in Healthy Laminae (CNTRL, *n* = 1), Acute Laminitis (ALL, *n* = 1), and Chronic Laminitis (CLL, *n* = 1). Each analysis was done in three independent experiments. The results are presented as ΔCt = (Ct Mean RG – Ct IG). For each gene, mean ± SD are indicated by horizontal bars.

For gene expression analysis to validate the receptor expression study, total RNA extraction was carried out using a RNeasy FFPE Kit (Qiagen Hilden, Germany) with a few modifications, as described by Rodrigo Zamith Cunha (17). Randomization was made with Artificial Intelligence (Chat GTP-4). After spectrophotometric quantification, the total RNA (1,000 ng) was reverse transcribed to cDNA using 5X iScript RT Supermix (Bio-Rad Laboratories Inc., Hercules, CA, United States) at a final volume of 20 μL. To evaluate the gene expression profiles, RT-PCR was carried out in a CFX96 thermal cycler (Bio-Rad Laboratories Inc.) using SYBR green detection to target the genes. RT-PCR was performed by using equine specific (15, 17) to evaluate the gene expression for Cannabinoid receptors 1 and 2 (*Cn1r* and *Cn2r*), and G protein-coupled receptor 55 (*GPR55*). Regarding the reference genes, *GAPDH* (Glyceraldehyde-3-phosphate dehydrogenase), *HPRT* (Hypoxanthine phosphoribosyltransferase 1) and *β-Act* (beta Actin) were based on horse sequences as previously reported (31). All the amplification reactions were carried out in 20 μL as previously described (17). The specificity of the amplified PCR products was confirmed by agarose gel electrophoresis and melting curve analysis. The relative expression of the genes of interest (IGs) was normalized based on the geometric mean of the three reference genes (RGs). The relative mRNA expression of the genes tested was evaluated using the ΔCt method with ΔCt = (Ct geometric mean ref. genes – Ct interested gene) which directly correlated with the expression level (ΔCt values very negative, lower expression; ΔCt values less negative higher expression) (Figure 1).

### 2.7 Western blotting

Tissue collection and WB procedure were conducted following Galiazzo et al. (32) and Chiocchetti et al. (33). Briefly: lamellar sample was collected, frozen in liquid nitrogen and stored at −80 °C until sample processing. Following, 100 mg of tissue was homogenized in 1 mL of SDS buffer (Tris–HCl, 62.5 mM; pH 6.8; SDS, 2%; and glycerol, 20%) and supplemented with a protease inhibitor cocktail (Sigma-Aldrich, Co, St. Louis, MO, USA). Total protein content was determined by Peterson’s Modification of Lowry Method using a Protein Assay Kit. An amount of 20 μg of total proteins were separated with NuPage4–12% bis-Tris Gel (Life Technologies Ltd., Paisley, United Kingdom) for 30 min at 200 V and then using electrophoresis transferred onto a nitrocellulose membrane with a semi-dry system (Trans Turbo Blot Bio -Rad). Non-specific binding on nitrocellulose membranes was blocked with 5% milk powder in PBS-T20 (Phosphate Buffer Saline-0.1% Tween-20) for 1 h at room temperature. After blocking treatment, the membrane was incubated overnight at 4 °C first with the primary antibodies (GPR55) 1:500 diluted in PBS with 1% of milk, and then with an appropriate dilution of biotinylated secondary antibody. Immunoreactive bands were visualized using chemiluminescent substrate (Clarity Western ECL Substrate Bio Rad), according to the manufacturer’s instructions. The intensity of the luminescent signal was acquired by Chemidoc Instrument (Bio Rad) and the apparent molecular weight of the resultant bands was analyzed by Quantity One Software (Bio-Rad).

**Figure 2:**
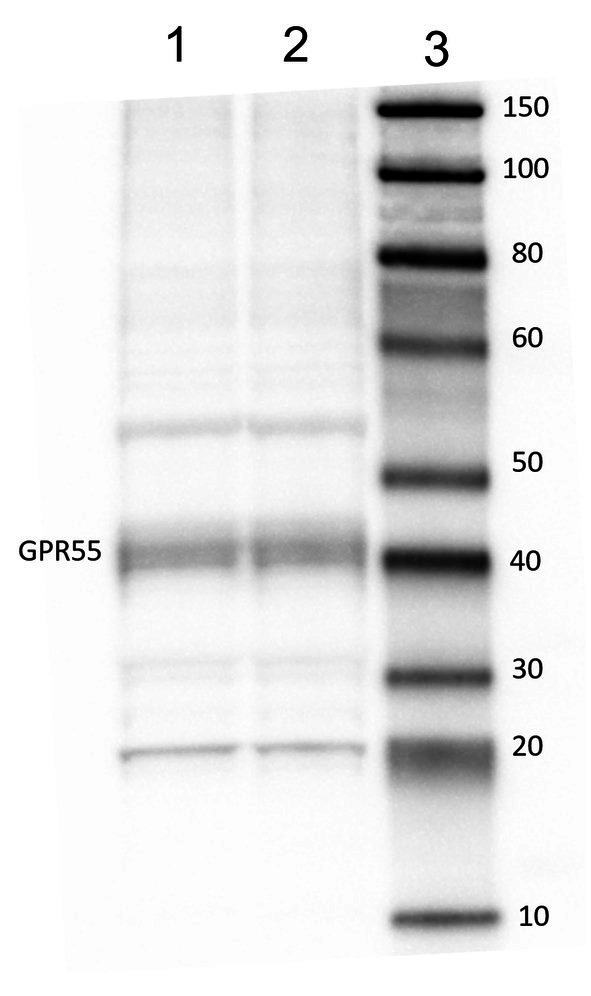
Representative image of Western Blot analysis showing the specificity of anti GPR55 antibody. Lane 1, 20 ug of CNTRL laminae, lane 2, 20 ug of CNTRL laminae, lane 3, Molecular weight markers (kDa).

Western blot analysis of GPR55 revealed a stronger band of ~ 40 kDa (Figure 2).

The primary antibody anti-CB1R (ab23703) utilized in current study had been validated and tested on horse tissue using WB analysis (13).

The rabbit anti-CB2R antibody (PA1-744) utilized in the current study had already been tested using WB analysis on horse tissue (34).

### 2.8 Immunohistochemistry on cryosections

Cryosections were hydrated in PBS. Endogenous peroxidase was blocked by immersion in 3% H_2_O_2_ in methanol for 30 min at room temperature (RT) (22–25 °C) and were then rehydrated. The blocking of non-specific antigenic sites was achieved by incubating the slides in Blocking solution (PBS with 3%of bovine serum albumin (Sigma Aldrich, Milan, Italy, Europe) and 0.25% Tween20 (Sigma Aldrich, Milan, Italy, Europe)) for 30 min at RT and then incubated overnight in a humid chamber at 4 °C with the primary antibodies diluted in blocking solution at the dilutions as described in Table 3. The slides were rinsed in TRIS buffer and subsequently incubated with a secondary biotinylated antibody (Vector Laboratories, Burlingame, CA, United States) diluted 1:200 in blocking solution. Following two additional washes in TRIS buffer, immunoreactivity was revealed using the avidin-biotin immunoperoxidase method (Vectastain Elite ABC Kit, Vector Laboratories, Burlingame, CA, United States) and visualized with the chromogen 3,3′-diaminobenzidine (0.05% w/v, cat# ACB999, Histo-Line Laboratories, Pantigliate, MI, Italy). The slides were then counterstained with Harris hematoxylin (cat# 01HEMH2; Histo-Line Laboratories) and permanently mounted using DPX medium (Fluka, Riedel-de Haen, Germany). Image acquisition was performed with an optical microscope (Eclipse E600; Nikon, Shinjuku, Japan) equipped with a USB 3.0 camera from the Imaging Source “33” Series (cat# DFK 33UX264; Bremen, Germany).

**Table 3:** Primary antibodies used in the study.

Primary antibodies	Host	Code	Diluition	Source
CB1R	Rabbit	ab23703	IF 1:100 IHC 1:300	Abcam
CB2R	Rabbit	PA1-744	IF 1:250 IHC 1:400	ThermoFisher
CB2R	Mouse	sc-293188	IF 1:50	Santa Cruz
GPR55	Rabbit	NB110-55498	IF 1:200 IHC 1:400	Novus Biol.
IBA1	Goat	NB100-1028	IF 1:80	Novus Biol.
CD3	Rat	CD3-12	IF 1:30	Leucocyte’s Antigen Laboratory, UC Davis
MAC387	Mouse	M0747 Clone MAC387	IF 1:400	Dako
Vimentin	Mouse	IS630 Clone V9	IF 1:600	Dako
Substance P	Rat	10-515A	IF 1:500	Fitzgerald

Primary antibody suppliers: Abcam, Cambridge, UK; Alomone, Jerusalem, Israel; Biorbyt Ltd., Cambridge, UK; Fitzgerald Industries Int., Inc. Concord, MA, USA; Novus Biologicals, Littleton, CO, USA; Thermo Fisher Scientific, Waltham, MA, USA; Dako Cytomation, Golstrup, Denmark. IF, immunofluorescence; IHC, immunohistochemistry.

### 2.9 Immunofluorescence on cryosections

The cryosections from the 18 horses were hydrated in PBS and processed for immunostaining. To block non-specific bindings, the sections were incubated in a solution containing 20% normal donkey serum (Colorado Serum Co., Denver, CO, USA), 0.5% Triton X- 100 (Sigma Aldrich, Milan, Italy, and Europe) and bovine serum albumin (1%) in PBS for 1 h at room temperature (RT) (22–25 °C). The cryosections were incubated in a humid chamber overnight at RT with the antibodies directed against CB1R, CB2R and GPR55 (single immunostaining) or with a cocktail of primary antibodies (double immunostaining) (Table 3) diluted in 1.8% NaCl in 0.01 M PBS containing 0.1% sodium azide. After washing in PBS (3 × 10 min), the sections were incubated for 1 h at RT in a humid chamber with the secondary antibodies (Table 4) diluted in PBS. Laboratory procedures followed as described (16, 35). To identify macrophages, the anti-ionized calcium binding adapter molecule 1 (IBA1) antibody was employed. In addition, the antibody anti-MAC387 (calprotectin) identified neutrophils (36–38) To identify the fibroblasts, the antibody anti-vimentin (Clone V9) was used (39). To identify the lymphocytes T, the antibody anti-CD3 was used. To identify afferent peptidergic nervous fibbers, the antibody anti-substance P (SP) was used.

**Table 4:** Secondary antibodies used in the study.

Secondary antibodies	Host	Code	Diluition	Source
Anti-mouse IgG Alexa-594	Donkey	A-21203	1:500	Thermo Fisher
Anti-rabbit IgG Alexa 488	Donkey	A-21206	IF 1:1000	Thermo Fisher
Anti-rabbit biotinylated	Goat	BA-1000	IHC 1:200	Vector laboratories
Anti-Goat IgG 594	Donkey	ab150132	IF 1:600	Abcam
Anti-Rat IgG Alexa 594	Donkey	A-21209	IF 1:500	Thermo Fisher

Secondary antibody suppliers: Abcam, Cambridge, UK; Thermo Fisher Scientific, Waltham, MA, USA; Vector Laboratories, Newark, CA, USA.

The preparations were examined using a Nikon Eclipse Ni microscope equipped with the appropriate filter cubes, and the images were recorded using a DS-Qi1Nc digital camera and NIS Elements software BR 4.20.01 (Nikon Instruments Europe BV, Amsterdam, The Netherlands, Europe). Slight brightness and contrast adjustments were made using Corel Photo Paint, whereas the figure panels were prepared using (Corel Draw Mountain View, Ottawa, ON, Canada).

### 2.10 Specificity of the primary antibodies

The specificity of the primary antibody anti-CB1R (ab23703) utilized in current study had been validated and tested on horse tissue using WB analysis (13).

The rabbit anti-CB2R antibody (PA1-744) utilized in the current study had already been tested using WB analysis on horse tissue (34). In this study, another anti-CB2R antibody, raised in mouse serum (sc-293188), was also used, whose specificity has not been tested on horse tissue; however, both the mouse and rabbit anti-CB2R antibodies were tested in a double-staining protocol and were co-localized in horse tissue (Supplementary Figure 2).

The rabbit anti-GPR55 antibody (NB110-55498) utilized in the current study has already recently been used on horse tissue (15). The immunogen used to obtain the anti-GPR55 antibody was a synthetic 20 amino acid peptide from the third cytoplasmic domain of Human GPR55 in amino acids 200–250. The homology between the full amino acid sequences of the horse and human GPR55 was 80%, and the correspondence with the specific sequence of the immunogen was 78%.^1^ The specificity of this antibody was tested in the present study.

The mouse anti-calprotectin (CAL) (clone MAC387), a complex of the mammalian proteins S100A8 and S100A9 (S100A8/A9), was recently shown to only label neutrophils on canine skin (40). The observation of Dapi-labeled multilobed nuclei confirmed that the CAL immunoreactive cells in the lamellar dermis of the horse were neutrophils.

The goat anti-IBA1 antibody, recently used on horse tissue (14, 15) was directed against a peptide having the sequence C-TGPPAKKAISELP, from the C Terminus of the porcine IBA1 sequence. Horse and porcine IBA1 molecules share 92.3% identity (see text footnote 1, respectively). It has been already used to identify macrophage and macrophage-like cells in horse tissue (15).

The rat anti-CD3 antibody was used to label the T lymphocytes in horses. CD3-12 is a rat-derived monoclonal antibody that recognizes the human CD3ε protein, a component of the T-cell receptor (TCR) complex essential for T-cell activation and development. Specific staining of equine T cells in paraffin-embedded sections has been demonstrated (41). The UniProt entry for the CD3 epsilon (CD3ε) chain, the target of the CD3-12 antibody, is P07766; the target for equine gene is A0A9L0RAV5_HORSE. Equine and Human CDε molecules shares 64.7% similarity (see text footnote 1, respectively). Staining with an antibody against the same protein has been demonstrated in horses (42).

The specificity of the rat anti-SP antibody was previously tested on horse tissue (43).

The mouse anti-vimentin (Clone V9) antibody has already been used to label fibroblasts in the horse skin (16, 44).

### 2.11 Specificity of the secondary antibody

The specificity of the secondary antibodies was tested by applying them to the sections after the omission of the primary antibodies (17). No stained cells were detected after omitting the primary antibodies.

### 2.12 Immunofluorescence and immunohistochemistry qualitative and semi-quantitative analysis

The immunoreactivity of the antibodies was evaluated, and their cellular localization (cytoplasmic, membranous, and nuclear) was reported. Five different slides were evaluated for each horse on which at least three random areas were evaluated. The intensity of expression was assessed semi-quantitatively in images acquired with consistent exposure times, categorized as faint, moderate, or bright. The proportions of cells immunoreactive for markers of macrophages/DCs (IBA1), T-cells (CD3), and neutrophils (calprotectin/MAC387), which were also immunoreactive for one of the cannabinoid receptors (CB1R, CB2R, GPR55), were determined by examining fluorescently labeled, double-stained preparations. A total of thirty (*n* = 30) cells from each animal were counted for each inflammatory cell marker. The percentage of immunoreactive cells for each specific marker (IBA1, CD3, CAL), and immunoreactive for a cannabinoid receptor (CB1R, CB2R, GPR55) was calculated and expressed as mean ± standard deviation. The data analysis was carried out in duplicate by two different observers.

### 2.13 Statistical analysis

The number of inflammatory cells immunoreactive for each specific marker (IBA1, CD3, CAL), and immunoreactive for a cannabinoid receptor (CB1R, CB2R, GPR55) was calculated and expressed as a percentage. Data are presented as mean ± standard deviation (SD) for normally distributed variables and as median with interquartile range (IQR) for non-normally distributed variables. The normality of the data was assessed using both the Shapiro–Wilk and the Kolmogorov–Smirnov tests. The normal data were analyzed using an ordinary one-way analysis of variance (ANOVA) followed by the Tukey’s Multiple Comparison Test; The non-normal data were analyzed using the non-parametric Kruskal-Wallis’s test followed by the Dunn’s Multiple Comparison Test, with a 95% CI and a significance level of *p* < 0.05. Data analysis was carried out in duplicate by two different researchers. The statistical analyses were carried out using GraphPad Prism software.

## 3 Results

### 3.1 Immunohistochemistry on cryosections

**Figure 3:**
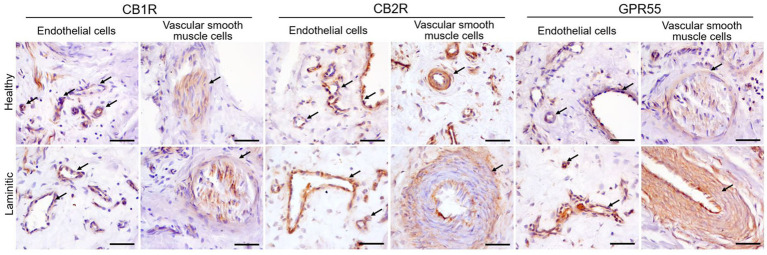
Representative immunohistochemical images on cryosections of HL and LL for anti-CB1R, -CB2R and -GPR55 antibodies. The arrows indicate endothelial cells and vascular smooth muscle cells positive for each respective cannabinoid receptor. Scale bar 50 μm.

*Cannabinoid Receptor 1 -* Moderate CB1R immunoreactivity (CB1R-IR) was observed in the cytoplasm of the endothelial cells of the blood capillaries in health and in laminitic primary dermal lamellae (PDL) and secondary dermal lamellae (SDL). Vascular smooth muscle cells also showed occasional faint positivity for CB1R (Figure 3). Inflammatory cells were negative for CB1R.

**Figure 4:**
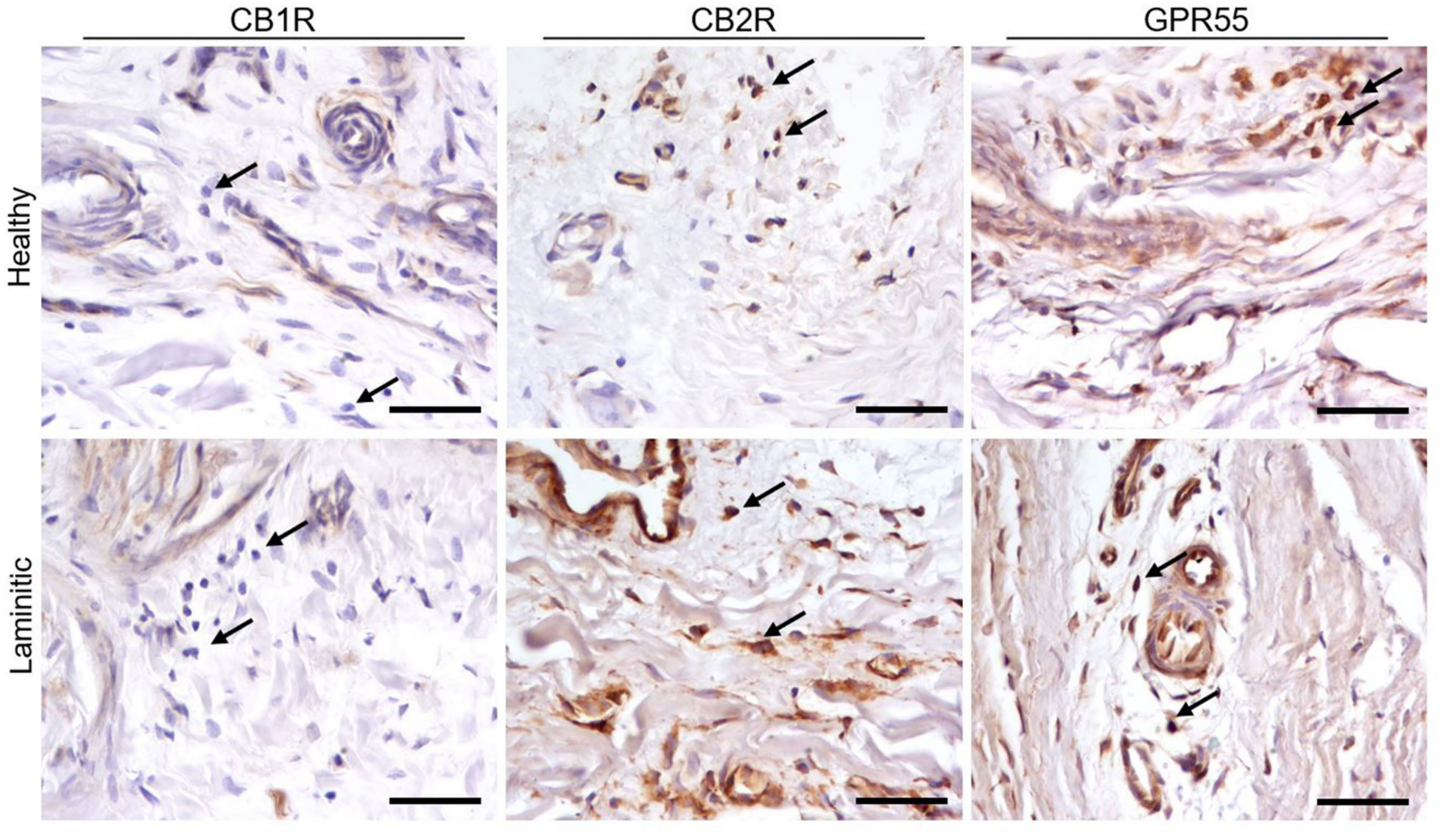
Representative immunohistochemical images of cryosections from HL and LL, stained with anti-CB1R, -CB2R and -GPR55 antibodies. The arrows highlight the inflammatory cells which exhibit the expression of CB2R and GPR55, but not of CB1R. Scale bar 50 μm.

*Cannabinoid Receptor 2 -* In the HL group, the cytoplasm of the endothelial cells of the PDL and the SDL blood vessels showed moderate expression of CB2R-IR; in the LL it showed bright expression. The cytoplasm of the vascular muscle cells showed moderate expression of CB2R (Figure 3). Cannabinoid receptor 2 was strongly expressed in the inflammatory cells present in the LL, whereas its expression was moderate in the inflammatory cells of the HL group. (Figure 4).

*G-Protein Coupled Receptor 55 -* A moderate GPR55-IR was observed in the cytoplasm of the blood capillary endothelial and vascular smooth muscle cells in the healthy PDL and the SDL. In contrast, in the same cells of laminitic PDL and SDL, the expression of GPR55 was brighter (Figure 3); GPR55 was strongly expressed in the inflammatory cells of both the HL and LL groups (Figure 4).

### 3.2 Immunofluorescence on cryosections

#### 3.2.1 Cannabinoid receptor 1

**Figure 5:**
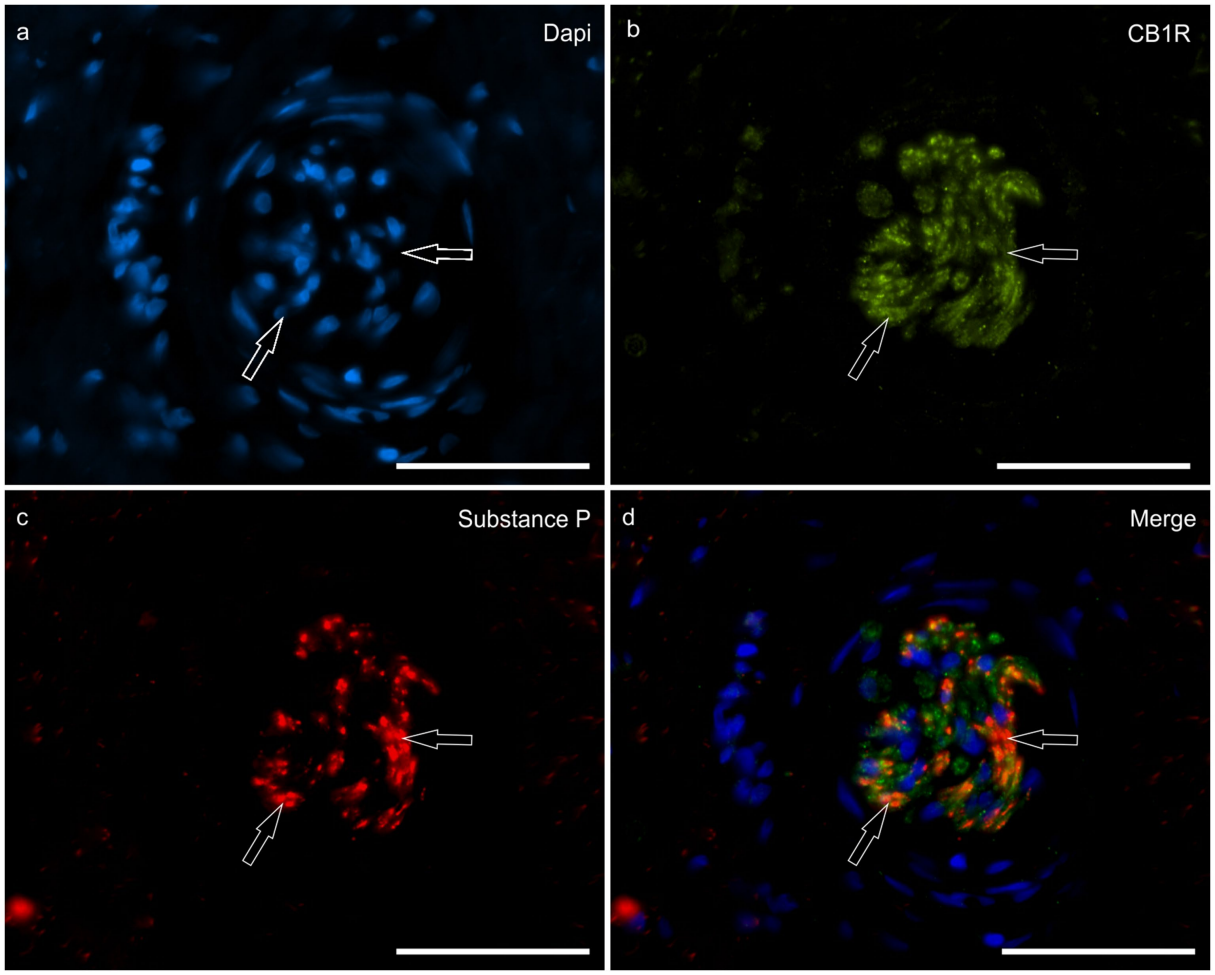
Photomicrographs of the laminar junction showing cannabinoid receptor 1 (CB1R) and Substance P (SP) immunoreactivity in the nerve fibers of the equine hoof. **(a–d)** the open arrows indicate the co-localization between CB1R- and SP-IR at the nerve fibers of the neurovascular plexus of the laminar junction. Scale bar: 50 μm.

*HL group -* There was no CB1R immunolabeling on the inflammatory cells, fibroblasts or endothelial cells in the HL group. Nerve fibers of the dermis co-expressed CB1R- and SP-IR in all the horses (100%) (16/16) (Figure 5).

*LL group* – The endothelial cells and fibroblasts of 25% (1/4) of the horses of the ALL group and 38% (3/8) of the CLL group expressed faint to moderate CB1R-IR.

#### 3.2.2 Cannabinoid receptor 2

**Figure 6:**
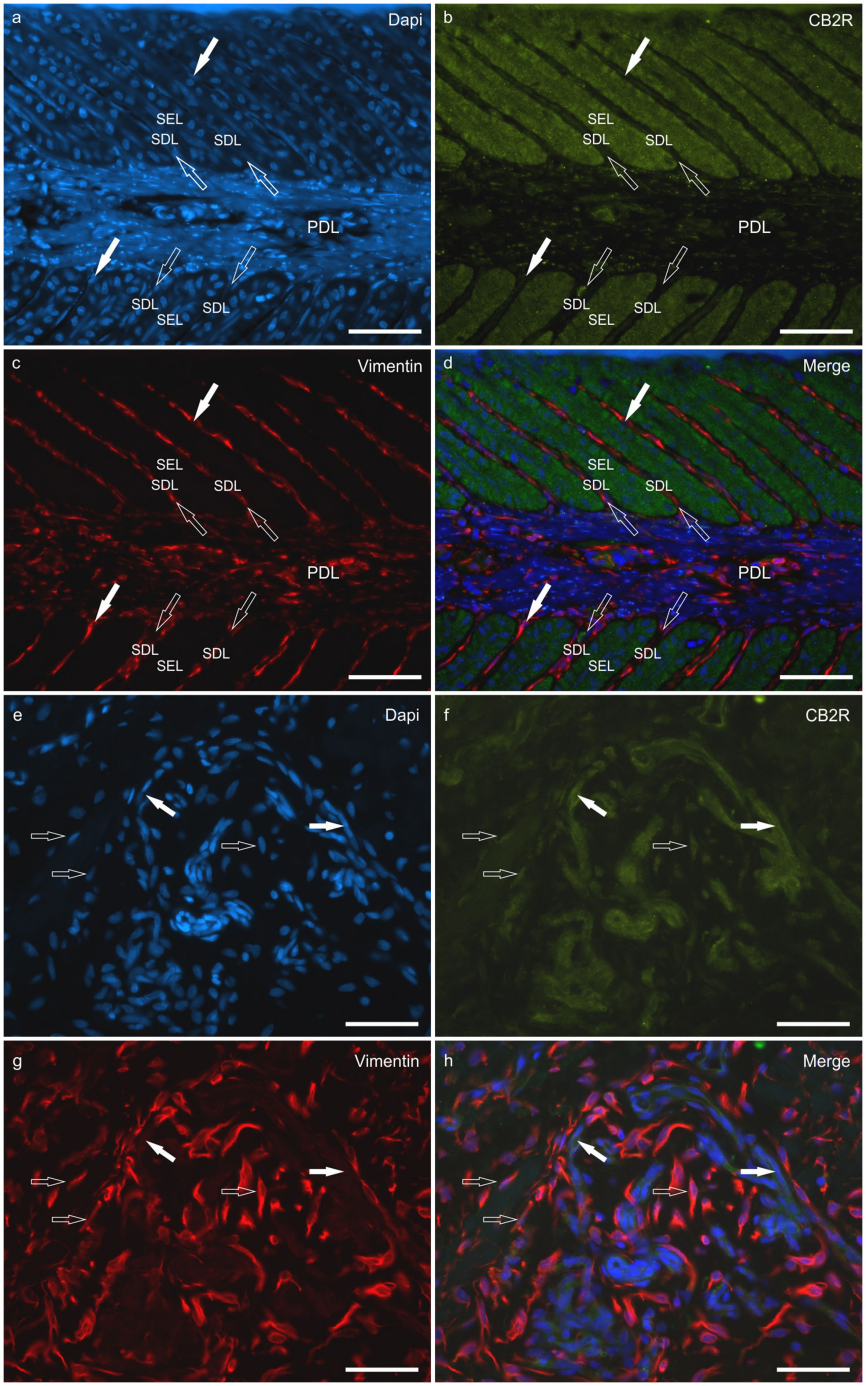
Photomicrographs of the laminar junction of the equine hoof showing the co-localization between the anti-cannabinoid receptor type 2 (CB2R) antibody and the anti-vimentin antibody which is a marker for fibroblasts **(a–h)**. **(a–d)** The epithelial cells of the secondary epidermal lamellae (SEL) showed bright CB2R immunoreactivity; the white arrows indicate the fibroblasts in the secondary dermal lamellae (SDL) showing bright vimentin immunoreactivity. The fibroblasts were also abundant in the primary dermal laminae (PDL). **(e–h)** The white arrows indicate vascular endothelial cells positive for CB2R; the open arrows indicate fibroblasts co-expressing vimentin and CB2R immunoreactivity. Bar: 50 μm.

**Figure 7:**
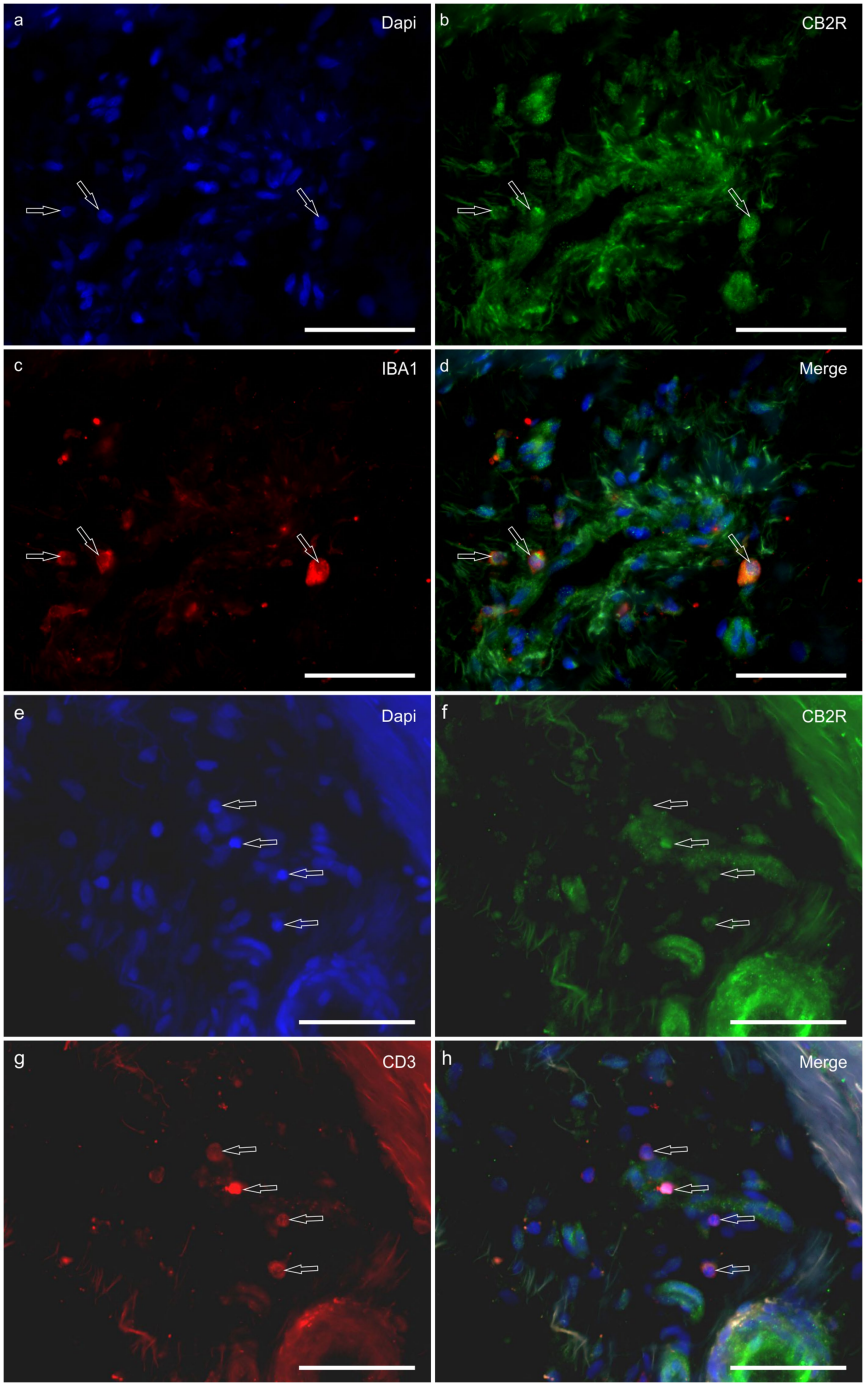
Photomicrographs of the laminar junction of the equine hoof showing the co-localization between the anti-cannabinoid receptor type 2 (CB2R) antibody and the anti-IBA1 antibodies (marker for macrophages/dendritic cells) **(a–d)** and anti-CD3 (marker for T cells) **(e-h)**. The open arrows indicate the Dapi-labeled nuclei of CB2R immunoreactive cells which were also IBA1 positive **(a–d)** and CD3 positive **(e–h)**. Bar: 50 μm.

**Figure 8:**
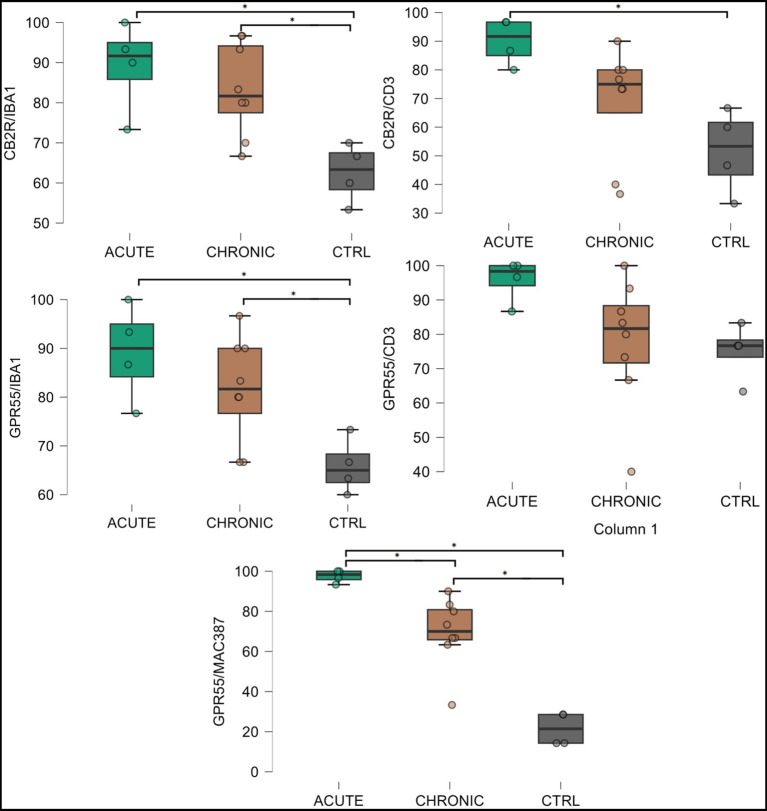
The graph illustrates the expression of cannabinoid receptors CB2 and GPR55 in different inflammatory cell markers (CD3, IBA1 and MAC387) across three groups: healthy laminae (CTRL), acute laminitis (ACUTE) and chronic laminitis (CHRONIC). The y-axis represents the percentage of cells expressing the respective cannabinoid receptors. Error bars indicate the standard error deviation and mean.

*HL group -* In the HL group, 100% of the horses (4/4) showed fibroblasts, vimentin-positive cells and vascular endothelial cells expressing moderate-to-bright cytoplasmatic CB2R-IR (Figure 6). Moderate CB2R-IR was also expressed by the cytoplasm of the macrophages/DCs and T-cells (Figure 7) present inside the blood vessels and around the neurovascular plexus within the deep dermis. The percentage of CD3 and IBA1 cells positive for CB2R were 52 ± 14.7% and 63 ± 7.3%, respectively (Figure 8).

*LL group* - In the ALL and CLL groups, the macrophages/DCs and T cells present in the inflammatory infiltrate and inside the blood capillaries of the dermis/PEL/SEL were brightly labeled for CB2R. In addition, the endothelial cells of the blood vessels and the smooth muscle cells of the arteries showed bright CB2R-IR. In the ALL horses, the dermal fibroblasts showed bright CB2R-IR, whereas, in the CLL group, 50% of the horses (4/8) showed moderate to bright CB2R-IR, and 50% of the horses (4/8) showed faint CB2R-IR. The mean percentage and standard deviation of CD3 and IBA1 positive cells for CB2R in the ALL group were 90 ± 8.1% and 89 ± 11.3%, respectively (Figure 8). For the CLL group, the mean percentage and standard deviation of CD3 and IBA1 cells positive for CB2R were 69 ± 19.5% and 83 ± 11.5%, respectively.

There was no immunolabeling of CB2R by neutrophils.

#### 3.2.3 G-protein coupled receptor 55

**Figure 9:**
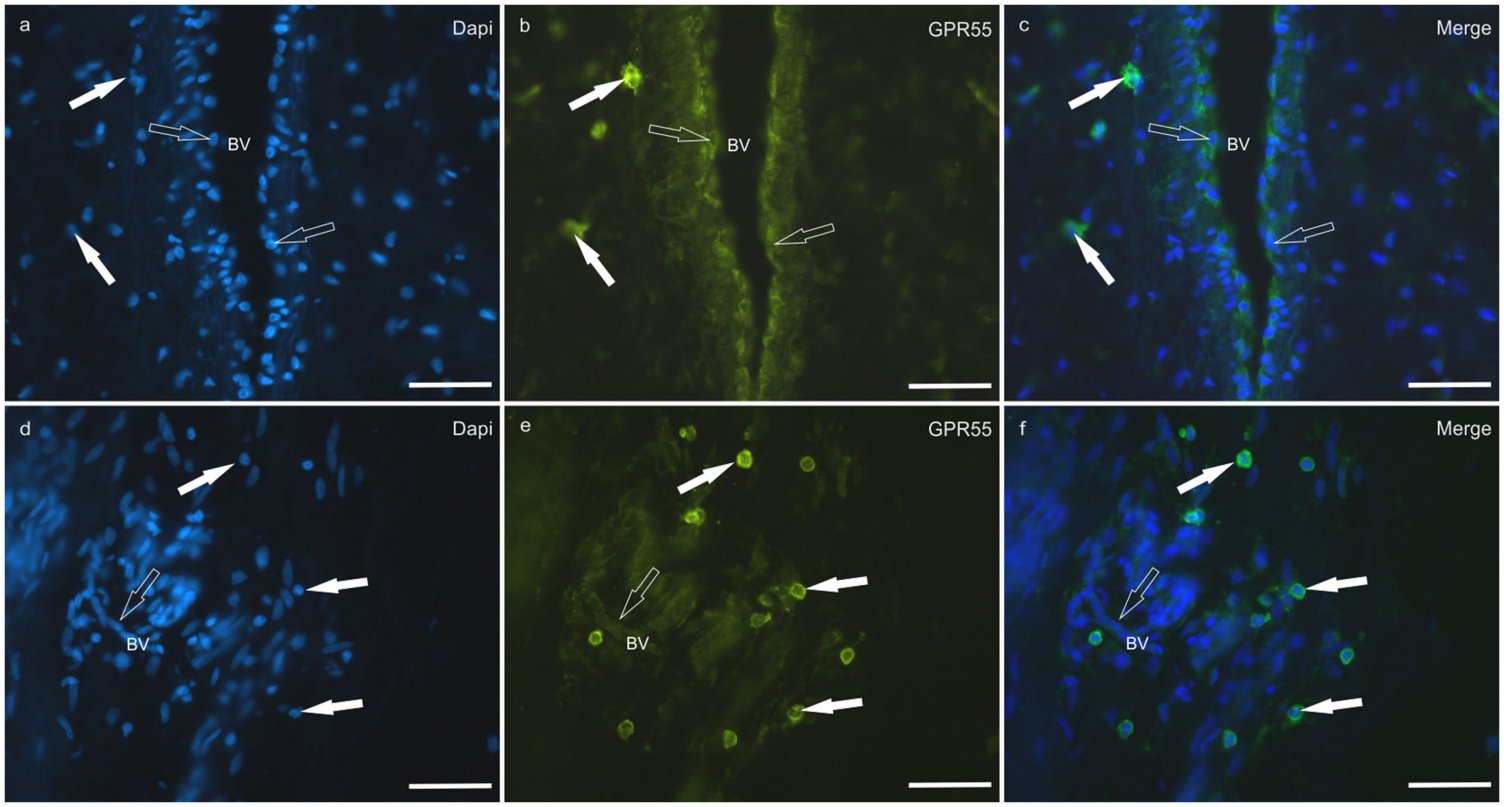
Photomicrographs of the laminar junction of the equine hoof. **(a–f)** Immunolabeling of the anti-G-Protein Coupled Receptor 55 (GPR55) antibody. The white arrows indicate the inflammatory cells around the blood vessels (BVs), while the open arrows indicate the endothelial cells positive for GPR55-IR. Bar: 50 μm.

**Figure 10:**
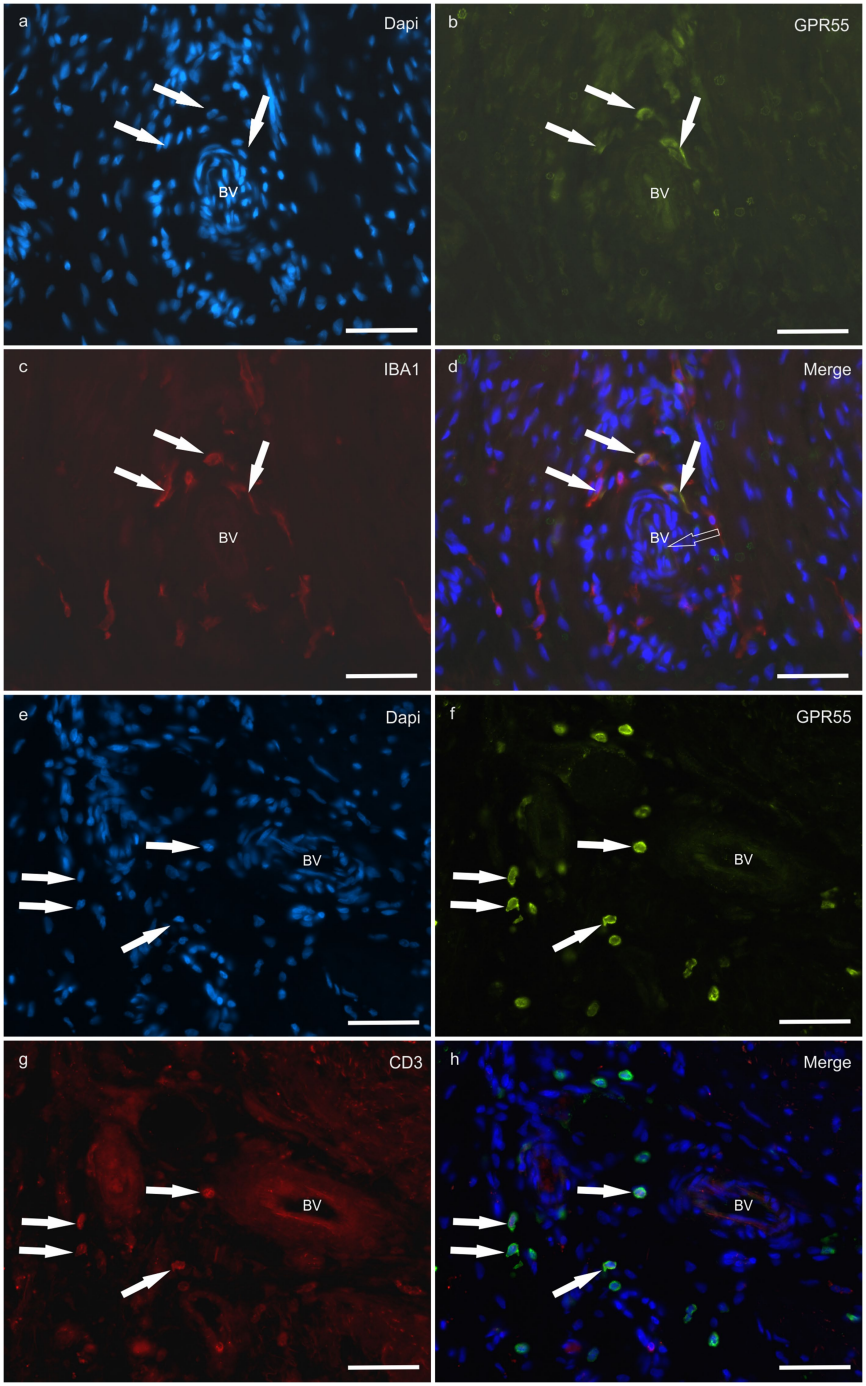
Photomicrographs of the laminar junction of the equine hoof. **(a–d)** Co-localization between the antibodies against the G-Protein Coupled Receptor 55 (GPR55) and macrophage marker IBA1 (white arrows) around a blood vessel (BV). **(e–h)** Co-localization between the cannabinoid-related receptor GPR55 and the antibody against T cells (CD3); the white arrow indicates the T cells positive for GPR55 around the blood vessels. Bar: 50 μm.

*HL group -* In the HL group, dermal cells showed cytoplasmatic GPR55-IR (Figure 9). The cytoplasm of vimentin positive fibroblasts and endothelial cells of blood vessels showed bright GPR55R-IR. The cytoplasm of macrophages/DCs, T-cells, and non-identified immune cells present inside blood vessels and around the neurovascular plexus within the deep dermis showed moderate to bright GPR55-IR (Figure 10). The percentage of CD3 and IBA1 positive cells which co-expressed GPR55-IR were 75 ± 8.3% and 66 ± 5.6%, respectively (Figure 8). Rare MAC387 positive cells (neutrophils) were observed in the HL group; it was not possible to count 30 cells. In total there were seven (7) neutrophils, 22 ± 8.7% positive for GPR55, all located inside the blood vessels of the deep dermis.

*LL group* - In the ALL group, the fibroblasts and the endothelial cells showed bright cytoplasmatic GPR55-IR. In the CLL group, the endothelial cells showed bright cytoplasmatic GPR55-IR; 63% (5/8) of the fibroblasts showed faint GPR55-IR, and 38% (3/8) showed moderate to bright GPR55-IR. In both groups, bright cytoplasmatic GPR55-IR was expressed by the macrophages/DCs, neutrophils, T-cells and non-identified immune cells of the inflammatory infiltrate and inside the blood capillaries of the dermis/PEL/ SEL. In the ALL group, the percentage of CD3, IBA1 and MAC387 positive cells which co-expressed GPR55-IR were 96 ± 6.3%, 89 ± 9.9%, and 98 ± 3.1%, respectively (Figure 8). In the CLL group, the mean percentage of CD3, IBA1, and MAC387 positive cells co-expressing GPR55 were 78 ± 18.6%, 82 ± 10.8% and 70 ± 17.3%, respectively (Figure 8).

### 3.3 Statistical analysis

*Cannabinoid Receptor 1 –* no analysis was carried out since none inflammatory cell was staining with CB1R antibody.

Cannabinoid Receptor 2 - The statistical analysis of the T cells positive for CB2R revealed a significant difference in T cell expression between the control and the acute laminitis groups (*p* = 0.0109). Comparisons between the control and the chronic groups (*p* = 0.4658), and between the acute and the chronic groups (*p* = 0.1579) were not statistically significant. Comparisons of the macrophages positive for CB2R revealed significant differences between the control and the acute laminitis groups (*p* = 0.0096), and between the control and the chronic laminitis groups (*p* = 0.0183).

*G-Protein Coupled Receptor 55 -* The statistical analysis of the T cells positive for GPR55 revealed no significant differences between the control and the acute laminitis groups (*p* = 0.1453), the control and the chronic laminitis groups (*p* = 0.9429) or the acute and the chronic laminitis groups (*p* = 0.1487). The statistical analysis of neutrophils positive for GPR55 revealed significant differences across all comparisons. There was a highly significant increase in GPR55 expression in the neutrophils between the control and the acute laminitis groups (*p* < 0.0001) as well as between the control and the chronic laminitis groups (*p* = 0.0002). In addition, a smaller but still significant difference was observed between the acute and the chronic laminitis groups (*p* = 0.0122). The statistical analysis of the macrophages positive for GPR55 revealed significant differences between the control and the acute laminitis groups (*p* = 0.0120), and between the control and the chronic laminitis groups (*p* = 0.0472); the difference between the acute and the chronic laminitis groups was not statistically significant (*p = 0.4378*).

## 4 Discussion

### 4.1 Laminae endocannabinoid system

The present protein expression confirmed by immunofluorescence and the presence of the gene for each protein evidenced by quantitative RT-PCR, provides confirmation of the distribution of novel pharmaceutical targets in the lamellar tissue. These findings are compatible with previous studies that showed cannabinoids receptors in the skin of horses (34), and in the epidermal cells of the hoof laminae (17). The G-protein coupled receptors of the ECS (type 1, type 2 and 55) are widespread in a compartmentalized way within the laminae. Its presence in different cell types and fluctuation across different disease stages suggests a pathophysiological role.

The Western Blot analysis of GPR55 revealed a major band of ~40 kDa, compatible with previous studies in other species (32, 45). Other faded bands were also noticed at ~20 kDa and ~55 kDa, this may be due to the existence of different heterodimers of this receptor. The G Proteins are made of 3 different sub-units: *γ*, *α* and *β* (46). The subunits α have a molecular weight between 40 to 45 kDa, while the subunits γ and β can have a molecular weight of ~37 to ~8 kDa (47). The receptor will change its structure depending on the signaling and binding. With the present result the antibody used is effective and specific for equine samples.

Accordingly, Kupczyk (34) found that for whole skin CBR1 has a molecular weight of ~50 kDa and CBR2 presented with two bands at ~70 kDa and ~40 kDa. After cell isolation, different bands were seeing. In keratinocytes CBR1 presented 3 bands, at ~50 kDa, ~55 kDa and ~33 kDa, while fibroblasts showed marked ~50 kDa and ~33 kDa bands; CBR2 presented with strong bands at ~70 kDa and ~40 kDa. These results holds in evidence the different subunits of the G-protein coupled receptors and align with the findings of the present study.

### 4.2 Endocannabinoid system role in inflammatory cells

Proteins of the ECS have being described in different species and its role on inflammatory regulation has been pointed (15, 16, 48, 49). Within the dermo-epidermal junction of the horse hoof, the macrophages, DCs, T cells, unidentified immune cells and blood capillaries strongly expressed CB2R and GPR55, while the neutrophils exclusively expressed GPR55. Previous studies have reported similar results in other species (36, 37).

The non-affected laminae analyzed in this study showed immunocytes inside the blood capillaries of the dermis (macrophages, lymphocytes and non-identified immune cells) strongly immunolabeled for CB2R and GPR55. Compatible with already described resident pool of lymphocytes and macrophages in the healthy laminae, which indicates that these leucocytes may play an important role in the early onset of laminar dysfunction and injury in laminitis (7, 50).

The macrophages present in the laminitic tissue in both the healthy and the laminitic hooves showed strong labeling for CB2R and GPR55, while the neutrophils present mostly in the laminitic horses were positive only for GPR55. Macrophages and fibroblasts produce IL-1β which is a pro-inflammatory cytokine and increases the expression of other inflammatory mediators including cicloossigenasi 2 (COX-2), IL-6, and matrix metalloproteinases, which are associated with the degradation and remodeling of tissue (51). In the present study, not only macrophages as also fibroblast were shown to be positive to CB2R and GPR55 at the normal and affected dermo-epidermal junction. A significantly increased migration of CD3 positive cells (T cells) into the lamellar junction was reported during the development and chronicity of laminitis (8), the present results showed strong CB2R and GPR55R immunolabeling in the same cells.

The significant differences observed in CB2R and GPR55 expressions in the neutrophils and macrophages during laminitis progression provide evidence that the ECS is actively involved in immune modulation. The higher number of neutrophils positive for GPR55 during the acute phase of the disease aligns with their well-established role (7, 52) in the early inflammatory response, reinforcing the fact that ECS components are engaged in immune cell recruitment and activation. As the disease progresses to a chronic stage, the reduction in neutrophil GPR55 expression and presence and the sustained increase in the macrophages showing CB2R- and GPR55-IR suggest a shift from an acute neutrophil-dominated response to macrophage-driven inflammatory regulation. Similarly, the significant increase in CB2R-positive T cells in acute laminitis suggests that T cells play an active role in modulating inflammation during the early phase of the disease. However, the lack of significant differences in GPR55 expressions in T cells across the different stages of disease indicates that its expressions do not depend on inflammatory stimuli. This dynamic receptor expression pattern indicates that the ECS plays a role in orchestrating immune cell behavior throughout different stages of laminitis. Furthermore, the persistent presence of CB2R- and GPR55-IR in both the resident immune cell pool and the inflammatory infiltrates highlights their role in immune regulation.

Supporting this hypothesis, a recent *in-vitro* study has shown that CBD at 4 μg/mL reduced the *in vitro* production of the inflammatory cytokines (TNF-*α* and IFN-*γ*) of the peripheral blood mononuclear cells from senior horses (53). Moreover, an *in-vivo* study involving senior horses pointed to a significant decrease in the inflammatory cytokine expression of IFN-γ for whole blood at day 60, and for IL-6 at days 60 and 90 for CBD-treated horses when compared to control horses (25), using an oil solution at a dose of 2 mg/kg orally for 90 days. Cannabinoids can actively suppress inflammation by downregulating the pro-inflammatory cytokines, such as IL-12, IL-6, IFN-γ, IL-8, IL-2, IL-1β, IL-15, and TNF-α (54), which are crucial pro-inflammatory molecules for the development of laminitis.

### 4.3 Endocannabinoid system role in fibroblasts

Evidence showed that the ECS can modulate the functioning of fibroblasts (55). Connective tissue and keratinocytes are known to remodel and continually upgrade their spatial organization by the tightly controlled production of a specific class of zinc-dependent enzymes MMPs that, when activated, degrade the extracellular matrix and basal membrane components (56). The major functions of MMPs are the remodeling and degradation of the extra-cellular matrix and cell membranes during various biological processes, such as cell migration and keratinocyte proliferation as well as angiogenesis (57).

In healthy equine hoof wall laminae, metalloproteinase −2 (MMP-2) and metalloproteinase-9 (MMP-9) had already been isolated (56), and they were shown to play key roles in the degradation of the BM, which consists mainly of type IV collagen. In human fibroblasts, it is known that MMP-9 and MMP-2 secreted from fibroblasts could play important roles in the tissue metabolism, including cytokine-induced inflammation (58). The regulatory effect of CBD on the level and activity of MMPs is known (59). The present finding of CB2R and GPR55 expression in fibroblasts is compatible with previous studies (34, 40). CB1R was expressed in the diseased group, although in both groups’ gene presence was validated. The activation of the CB1R leads to fibrogenesis, while the enhancement of the CB2R inhibits fibrosis progression (60).

### 4.4 Endocannabinoid system role in blood vessels

Reduced lamellar blood flow is a key event in the pathogenesis of laminitis, with ischemia and subsequent reperfusion resulting in lamellar damage. Parallelly, the same pathologic mechanisms which underlie the cardiovascular disease associated with human metabolic syndrome, including changes in insulin signaling, inflammatory cytokines, and vascular/endothelial dysfunction, could contribute to laminitis (8, 61, 62).

By means of CB2R pathways, endocannabinoid molecules, such as N-Oleoylethanolamine and 2-arachidonoyglycerol, can modulate the human endothelial – leukocyte interaction by reducing the release and production of adhesion molecules (63) and selectins (64), and CB2R may play a role in the vasodilatory function (65). Furthermore, GPR55 was shown to play a role in the influx of calcium in the endothelial cells (66); it plays a role in mechanical hyperalgesia associated with inflammation and neuropathic pain (67), and with cardiovascular function and heart failure (68).

In addition, CB1R inhibition leads to decreased vascular angiotensin II type 1 receptor (AT1R) expression, NADPH oxidase activity and ROS production *in vitro* and *in vivo*; this antioxidative effect is associated with improved endothelial function in apolipoprotein E-deficient (ApoE−/−) mice, indicating the beneficial direct vascular effects of CB1R inhibition (69).

### 4.5 Endocannabinoid System Role in nerve fibers

The present findings demonstrated the presence of CB1R in nerve fibers at the dermo-epidermal junction of the equine hoof. This aligns with the existing literature, showing that CB1R is localized in peptidergic nerve fibers (Substance P positive) which are involved in pain and inflammatory responses (13, 70, 71). Pre-clinical studies have shown the effective management of pain in horses using CBD and enhanced quality of life, with a dose ranging from 1 to 3 mg/kg (22, 25, 72). To the day, there are no studies which deal with controlling pain in horses with laminitis using cannabinoids as the drug of choice.

Despite our findings, several limitations must be acknowledged. The study included a relatively small and uneven number of samples across groups, which may limit the statistical power and generalizability of the results. Additionally, the absence of complete clinical histories for all animals, particularly those from abattoirs, hindered a more robust correlation between the etiology and severity of laminitis and the observed patterns of protein expression. The study design was not blinded, which could introduce potential observer bias during immunohistochemical analysis. Moreover, gene expression quantitative analysis was not performed for this study, nor did was investigate receptor functionality, intracellular signaling, or dynamic changes in protein expression over time. The heterogeneity in the underlying causes of laminitis could influence receptor distribution, and this was not stratified or analyzed separately in the current design.

## 5 Conclusion

The present findings highlight the presence of cannabinoid receptors CB1, CB2, and GPR55 in the inflammatory cells, fibroblasts and endothelial cells of healthy and pathological hoof lamellar epithelial tissue. The modulation of CB1R, CB2R, and GPR55 signaling pathways could offer novel therapeutic approaches for managing hoof diseases. Future research should focus on elucidating the precise mechanisms by which these receptors function within the hoof tissue, using larger cohorts of animals and examining correlations between cause, severity, chronicity and protein expression.

## Data Availability

The datasets generated and analyzed during the current study are available in the AlmaDL Institutional Repository, managed by the University of Bologna. Data can be accessed through the re3data-registered repository at https://doi.org/10.17616/R3P19R (ISSN: 2038-7954).

## Footnotes

1 https://www.uniprot.org/, accessed on 7 January 2025

## Supplementary material

Supplementary Figure 1 Sampling procedure scheme. 1: disarticulation of the front limbs; 2: 1st transactional cut with a band saw; 3: transversal view of the two pieces of the hoof after 1st cut; 4: 2nd and 3rd cuts with band saw, transversally to the axis of anatomical piece. 5: Photograph of the middle section after 2nd and 3rd cuts, full thickening sample of the laminae is dissected using bistoury. 6: laminae sample pre fixation.

Supplementary Figure 2 Co-localization between the rabbit anti-CB2R and mouse anti-CB2R antibodies.

## Glossary

ALL: Acute laminitic laminae
ANOVA: Analysis of variance
ApoE: apolipoprotein E
AT1R: Vascular angiotensin II type 1 receptor
BM: Basal membrane
BWE: Back-nut extract
CAL: Calprotectin
CBC: Complete blood count
CBD: Cannabidiol
CBGA: Cannabigerolic Acid
CB1R: Cannabinoid Receptor Type 1
CB2R: Cannabinoid Receptor Type 2
CLL: Chronic laminitic laminae
COX-2: Cicloossigenasi 2
DAPI: 6-diamidino-2-phenylindole
DC: Dendritic cells
DRG: Dorsal Root Ganglia
ECS: Endocannabinoid System
GPR55: G-Protein Coupled Receptor 55
HL: Healthy laminae
IBA1: Ionized calcium binding adapter molecule 1
ID: Insulin Dysregulation
IFN-*γ*: Interferon gamma
IL: Interleukins
IR: Immunoreactivity
LL: Laminitic laminae
MMP: Metalloproteinases
NaCl: Sodium Chloride
OCT: Optimal Cutting Temperature
PBS: Phosphate-buffered saline
PDL: Primary Dermal Laminae
PEA: Palmitoylethanolamide
PEL: Primary Epidermal Laminae
PPID: Pars Pituitary Intermedia Disfunction
ROS: Reactive oxygen species
RT: Room temperature
SDL: Secondary Dermal Laminae
SEL: Secondary Epidermal Laminae
SIRS: Systemic inflammatory response syndrome
SP: Substance P
THCV: Tetrahydrocannabivarin Acid
TNF-*α*: Tumor necrosis factor-α
Wb: Western blot
α: Alfa
γ: Gamma
