# Supplementary figures and images for: Cannabinoid and cannabinoid related receptors in fibroblasts, inflammatory and endothelial cells of the equine hoof with and without laminitis: novel pharmacological target
Source: Front Vet Sci. 2025 Nov 28;12:1723160. doi: 10.3389/fvets.2025.1723160 (PMC12699270; doi:10.3389/fvets.2025.1723160)

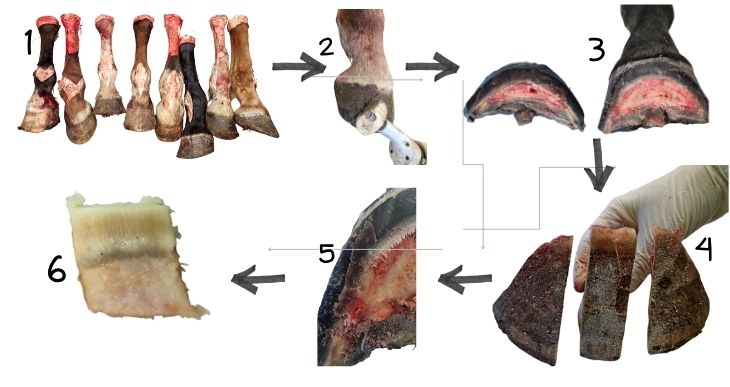

Supplement: Supplementary Figure 1 — Sampling procedure scheme. 1: disarticulation of the front limbs; 2: 1st transactional cut with a band saw; 3: transversal view of the two pieces of the hoof after 1st cut; 4: 2nd and 3rd cuts with band saw, transversally to the axis of anatomical piece. 5: Photograph of the middle section after 2nd and 3rd cuts, full thickening sample of the laminae is dissected using bistoury. 6: laminae sample pre fixation. [file Image_1.JPEG]

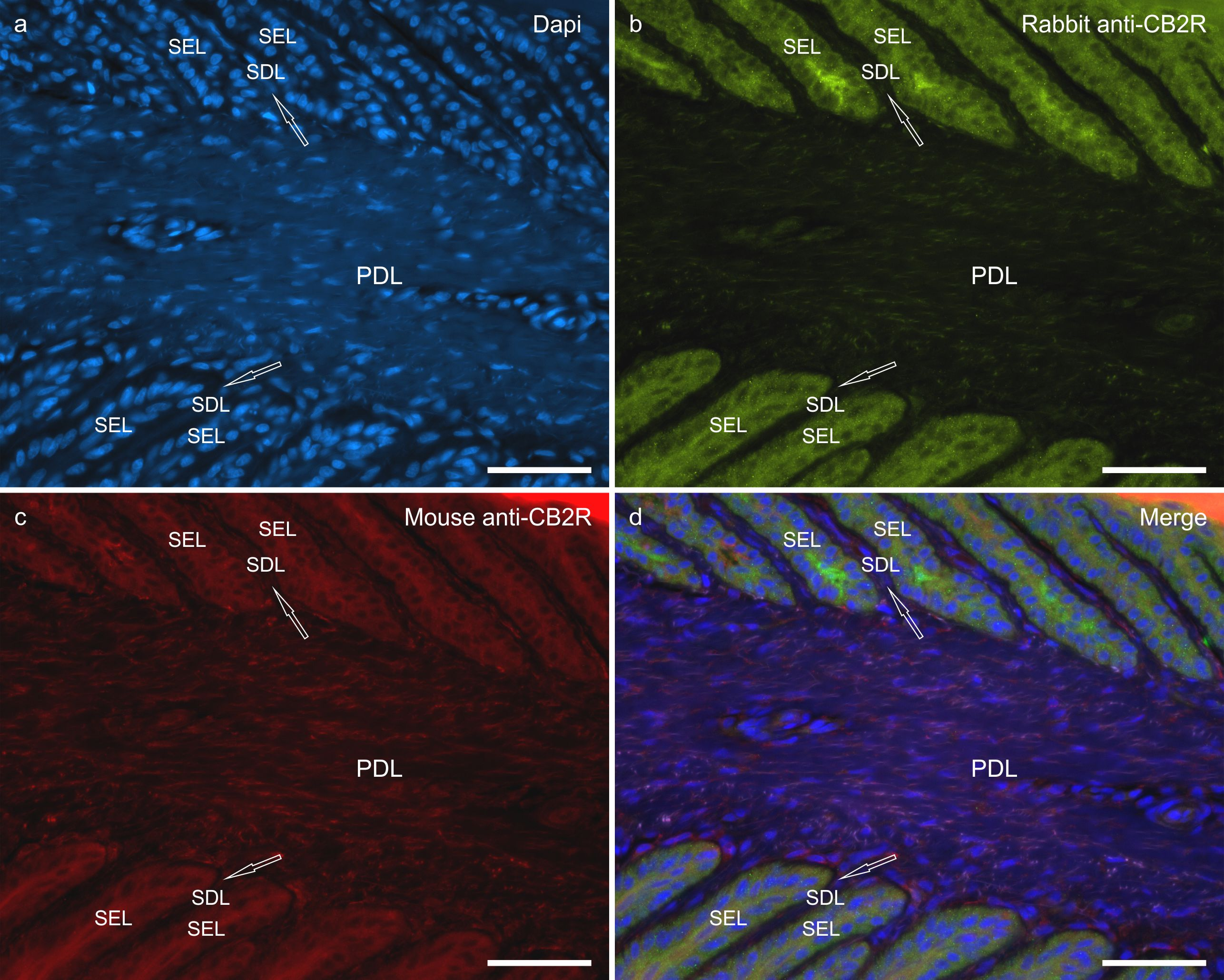

Supplement: Supplementary Figure 2 — Co-localization between the rabbit anti-CB2R and mouse anti-CB2R antibodies. [file Image_2.TIF]
